# *In Vitro* Efficacy of *Myxococcus fulvus* ANSM068 to Biotransform Aflatoxin B_1_

**DOI:** 10.3390/ijms11104063

**Published:** 2010-10-20

**Authors:** Shu Guan, Lihong Zhao, Qiugang Ma, Ting Zhou, Ning Wang, Xinxu Hu, Cheng Ji

**Affiliations:** 1 National Key Lab for Animal Nutrition, College of Animal Science and Technology, China Agricultural University, Beijing 100193, China; E-Mails: guanshu8@gmail.com (S.G.); lihongzhao100@126.com (L.Z.); maqiugang@cau.edu.cn (Q.M.); cauking@gmail.com (N.W.); huxinxu1980@126.com (X.H.); 2 Guelph Food Research Center, Agriculture and Agri-Food Canada, Guelph N1G5C9, Canada; E-Mail: zhout@agr.gc.ca

**Keywords:** aflatoxin B_1_, biotransformation, culture supernatant, Myxococcus fulvus ANSM068

## Abstract

Aflatoxin B_1_ (AFB_1_) is commonly found in cereals and animal feeds and causes a significant threat to the food industry and animal production. Several microbial isolates with high AFB_1_ transformation ability have been identified in our previous studies. The aim of this research was to characterize one of those isolates, *Myxococcus fulvus* ANSM068, and to explore its biotransformation mechanism. The bacterial isolate of *M. fulvus* ANSM068, isolated from deer feces, was able to transform AFB_1_ by 80.7% in liquid VY/2 medium after incubation at 30 °C for 72 h. The supernatant of the bacterial culture was more effective in transforming AFB_1_ as compared to the cells alone and the cell extract. The transformation activity was significantly reduced and eradicated after the culture supernatant was treated with proteinase K, proteinase K plus SDS and heating. Culture conditions, including nitrogen source, initial pH and incubation temperature were evaluated for an optimal AFB_1_ transformation. Liquid chromatography mass spectrometry (LCMS) analyses showed that AFB_1_ was transformed to a structurally different compound. Infrared analysis (IR) indicated that the lactone ring on the AFB_1_ molecule was modified by the culture supernatant. Chromatographies on DEAE-Ion exchange and Sephadex-Molecular sieve and SDS-PAGE electrophoresis were used to determine active components from the culture supernatant, indicating that enzyme(s) were responsible for the AFB_1_ biotransformation. This is the first report on AFB_1_ transformation by a strain of myxobacteria through enzymatic reaction(s).

## 1. Introduction

Aflatoxins are a group of structurally related secondary metabolites produced mainly by *Aspergillus flavus* and *Aspergillus parasiticus* [1]. Aflatoxin B_1_ (AFB_1_), one of the most hazardous mycotoxins, is extremely toxic, mutagenic and carcinogenic [2,3]. It poses a severe threat to both livestock productivity and human health, thus resulting in huge economic losses worldwide each year [4,5].

Interest in biological detoxification of AFB_1_ has greatly increased during the past decade. Several fungal species have been found to be able to biotransform AFB_1_ into less toxic metabolites; such fungi include *Pleurotus ostreatus* [6], *Trametes versicolor* [7], *Rhizopus* sp., *Mucor* sp. [8], and a few yeasts such as *Trichosporon mycotoxinivorans* [9], *Saccharomyces cerevisiae* [10], *Trichoderma* strains [11], and *Armillariella tabescens* [12]. The biotransformation activities of these fungi were mainly demonstrated with cell extracts. However, practical applications of these fungi may be limited by factors such as long incubation time required for the detoxification and complicated procedures needed for obtaining the active extracts. Reduction of AFB_1_ by bacteria has also been reported; most of the published studies focused on lactic acid bacteria, such as strains belonging to *Lactobacillus* [13,14], *Bifidobacterium* [15,16], *Propionibacterium* [17] and *Lactococcus* [18]. However, the AFB_1_ reduction by these bacteria was proven to be mainly by cell binding rather than biotransformation. Most importantly, this kind of binding seems to be reversible, which means that AFB_1_ can hardly be removed completely from contaminated media.

Recently, several studies reported biological transformation of AFB_1_ by microbial culture or their secondary metabolites. These microbes include *Rhodococcus erythropolis* [19], *Mycobacterium fluoranthenivorans* [20,21], *Stenotrophomonas maltophilia* [22] and *Nocardia corynebacterioides* (formerly *Flavobacterium aurantiacum*) [23–26]. Some studies further explored the role of enzymes in secondary metabolites on AFB_1_ biotransformation [6,27]. Evidence showed that AFB_1_ biotransformation by microorganisms and their metabolites, especially enzymes, is specific, effective and environmentally sound.

Myxobacteria are a kind of higher prokaryotic organism and are reported to be a rich source of secondary metabolites [28,29]. To date, more than 80 basic structures and 450 structural variants have been described from microbes, most of which are exclusively produced by myxobacteria [30]. One of the unique characteristics that myxobacteria possess is the prolific production of extracellular lytic enzymes and antibiotics, many of which are being used as human drugs [31–36]. It has been well studied that all myxobacteria specialize in biotransformation of biomacromolecules [30]. However, such microorganisms and their secondary metabolites have not been reported to show mycotoxin biotransformation ability. In our previous studies, several microbes with AFB_1_ biotransformation ability were isolated by using a newly developed coumarin medium method [22]. One of those microbes that was isolated from deer feces exhibited high AFB_1_ biotransformation ability in liquid culture. It was further identified as a bacterial strain of *Myxococcus fulvus* and designated *M. fulvus* K2 It was later renamed as *M. fulvus* ANSM068 according to the laboratory’s regulations.

The objective of this study was to further characterize *M. fulvus* ANSM068 as an AFB_1_ biotransformation agent, to optimize the biotransformation conditions and to explore the biotransformation mechanism.

## 2. Results and Discussion

### 2.1. AFB_1_ Biotransformation by *M. fulvus* ANSM068

**Figure 1:**
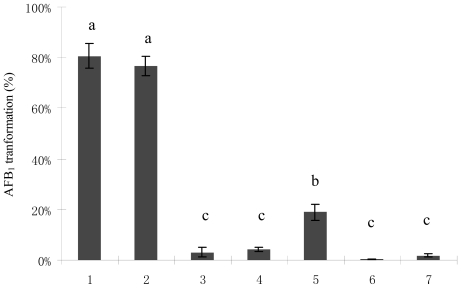
AFB_1_ biotransformation by *M. fulvus* ANSM068. 1. Liquid culture; 2. Culture supernatant; 3. Cell extracts; 4. Cell; 5. Proteinase K (Prok) treated culture supernatant; 6. Prok + SDS treated culture supernatant; 7. Heat treated culture supernatant. The reactions were performed at 30 °C for 72 h. Each value is a mean ± SE of three replicates. Means within a row with no common letters differ (P < 0.05).

The bacterial culture of *M. fulvus* ANSM068 reduced AFB_1_ by 80.7% after co-incubation at 30 °C for 72 h. The culture supernatant of *M. fulvus* ANSM068 was able to reduce AFB_1_ by 76.6%. However, no significant reduction was observed in the treatments with the cells and cell extracts (Figure 1). Culture supernatant treated with proteinase K (Prok) displayed significantly decreased reduction ability, from 76.6 to 18.9%. No reduction of AFB_1_ concentration was observed when the culture supernatant was treated with Prok plus SDS or with heating (boiling water bath for 10 min) (Figure 1).

**Figure 2:**
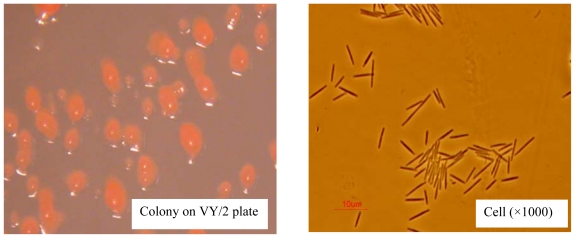
Colony (left) and cell (right) morphology of *Myxococcus fulvus* ANSM068.

Myxobacteria became well-known as prolific producers of secondary metabolites during the past few decades [37,38]. Thus far, no pathogenic myxobacteria have been observed and many bioactive components and drugs were isolated from myxobacteria [39–41]. *Myxococcus fulvus* was first documented and characterized in 1969 [42]. It is a species of myxobacteria, a group of Gram-negative eubacteria with rod-shaped cells. The colonial and cell morphology of *M. fulvus* ANSM068 are shown in Figure 2. It was previously found to be capable of reducing AFB_1_ [22]. Up to now, no study has focused on mycotoxin control by using myxobacteria. This is the first report on mycotoxin biotransformation by a bacterial strain belonging to myxobacteria.

Reduction of AFB_1_ by culture supernatant produced without pre-exposure to AFB_1_ suggested that it was achieved during the normal growth of the bacterium, indicating that the reduction was a constitutive activity of *M. fulvus* ANSM068. Results from proteinase K, proteinase K plus SDS and heating treatments implied that the reduction of AFB_1_ was due to biotransformation instead of cell binding. Protein or enzymes in culture supernatant might be involved in AFB_1_ biotransformation by the bacterial strain.

### 2.2. Optimal Culture Conditions for AFB_1_ Biotransformation

**Figure 3:**
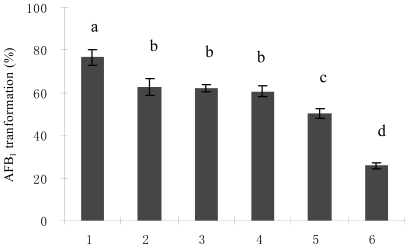
AFB_1_ biotransformation by *M. fulvus* ANSM068 in different nitrogen sources. 1. Yeast extract; 2. Peptone; 3. Beef extract; 4. Tryptone; 5. Fish peptone; 6. Ammonium nitrate. The reactions were performed at 30 °C for 72 h. Each value is a mean ± SE of three replicates. Means with no common letters differ (P < 0.05).

Nitrogen sources have a dramatic effect on AFB_1_ biotransformation by *M. fulvus* ANSM068. Yeast extract was found to be the best nitrogen source for AFB_1_ biotransformation and resulted in 76.6% AFB_1_ biotransformation (Figure 3). In comparison, AFB_1_ biotransformation was significantly (P < 0.05) lower with ammonium nitrate (25.8%). Among the four levels of yeast extract tested (0.2, 0.5, 0.8 and 1.2%), 0.5% was optimal for AFB_1_ biotransformation (76.6%). Nitrogen in substrates is one of the most important factors influencing myxobacteria growth and production of metabolites. Chun [43] studied the effect of different nitrogen sources on the production of antifungal metabolites by myxobacteria in liquid culture and found that yeast extract improved secretion of antifungal metabolites but fish meal, soybean powder and inorganic nitrogen treatments showed no function. A similar trend was also observed in this study. Yeast extract was most favored in AFB_1_ biotransformation by *M. fulvus* ANSM068 and inorganic nitrogen was far less effective. The reasons could be that in natural environments these myxobacteria feed on other organisms such as eubacteria or yeasts by bacteriolysis or cellular lysis [44]. The nutrients ratio such as amino acid and vitamins in yeast extract might be more suitable to the bacterial strain for its growth and production of AFB_1_ biotransformation components.

**Figure 4:**
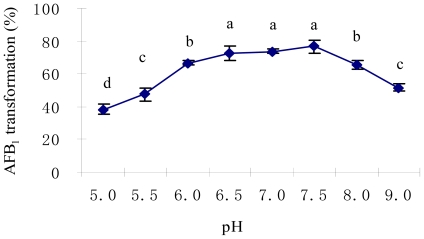
Effect of initial pH of the medium on AFB_1_ biotransformation by *M. fulvus* ANSM068. Initial pH value was determined by adjusting the culture pH to 5.0, 5.5, 6.0, 6.5 by using citrate acid buffer, and to 7.0, 7.5, 8.0, 9.0 by sodium phosphate buffer. The reactions were performed at 30 °C for 72 h. Each value is a mean ± SE of three replicates. Means within a row with no common letters differ (P < 0.05).

The initial pH value in culture medium showed a significant effect on AFB_1_ biotransformation (Figure 4). The highest AFB_1_ biotransformation rate was detected at pH values of 6.5 to 7.5 (P < 0.05). It has been well documented that the optimum pH range for myxobacteria is 6.8–7.8 in liquid culture [45]. Ahn *et al*. [46] studied the effect of pH value on antifungal metabolites produced by a *Myxococcus* isolate and found that a pH value between 6.5 and 7.6 was favored and the optimal pH value was 7.2.

**Figure 5:**
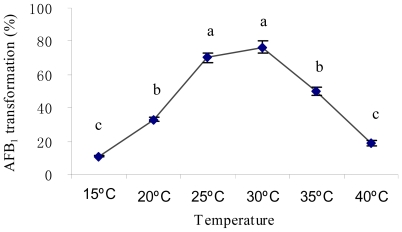
Effect of temperature on AFB_1_ biotransformation by *M. fulvus* ANSM068. The reactions were performed at 30 °C for 72 h. Each value is a mean ± SE of three replicates. Means within a row with no common letters differ (P < 0.05).

The effect of temperature on AFB_1_ biotransformation by *M. fulvus* ANSM068 is shown in Figure 5. The % biotransformation exhibited was significantly lower (P < 0.05) at 15 °C (11.0%) and 40 °C (18.9%) than at 30 °C (76.6%). Since the bacterial strain originated from deer feces, a temperature at 30 °C should be more suitable for the survival and growth of the bacterium, thus optimal for its enzyme system.

### 2.3. AFB_1_ Biotransformation Product Analysis

**Figure 6:**
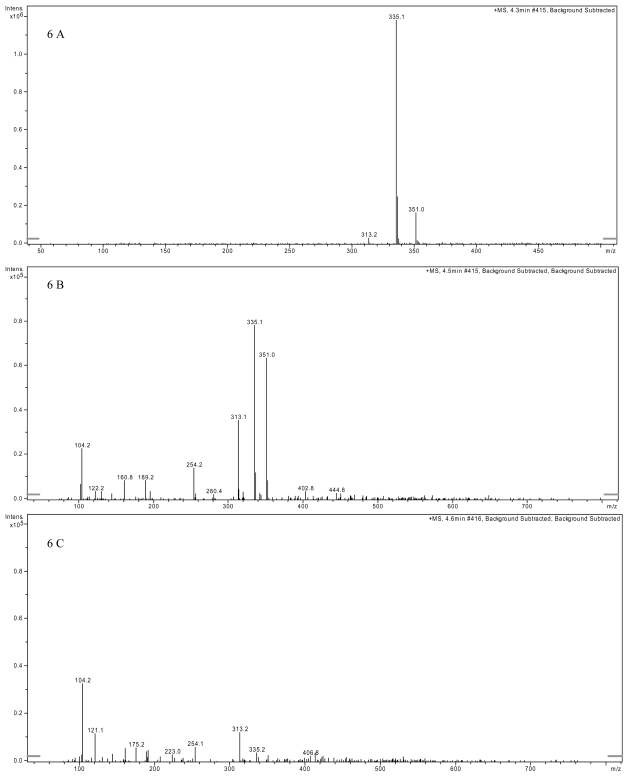
Detection of AFB_1_ by liquid chromatography mass spectrometry. (**A**) LC-MS spectrum of AFB_1_ standard at 100 ppb showing the distinct ions: [M + H]^+^ at m/z = 313, [M + Na]^+^ at m/z = 335 and [M + K]^+^ at m/z = 351. (**B**) LC-MS spectrum of VY/2 medium supplemented with AFB_1_ standard at 100 ppb. The three distinct ions can clearly be distinguished. (**C**) LC-MS spectrum of AFB_1_ after 72 h treatment with *M. fulvus* culture supernatant. The ion at 351 disappeared while the ions at 313 and 335 were significantly reduced.

In the analysis by LCMS, AFB_1_ standard was identified at m/z = 313 for the protonated cation [M + H]^+^, m/z = 335 for the sodium adduct of AFB_1_ [M + Na]^+^, and at m/z = 351 for the potassium adduct [M + K]^+^ (Figure 6A); VY/2 medium supplemented with AFB_1_ clearly showed the above three distinct ions (Figure 6B). However, the distinct AFB_1_ ion at m/z =351 disappeared while the ions at m/z = 313 and m/z = 335 significantly decreased after AFB_1_ was co-incubated with *M. fulvus* ANSM068 culture supernatant for 72 h (Figure 6C). These results suggest AFB_1_ level was significantly reduced although it was still present.

**Figure 7:**
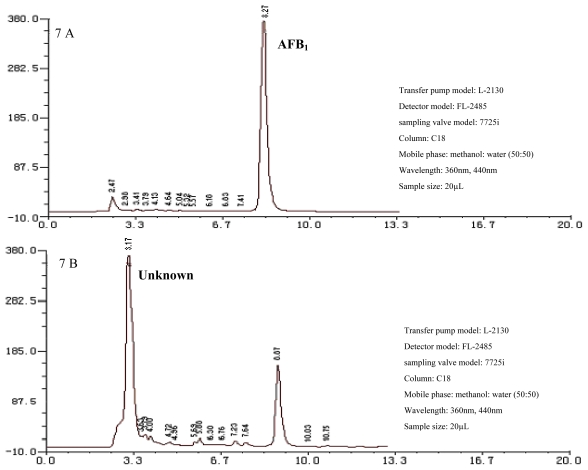
Detection of AFB_1_ biotransformation product by HPLC. (**A**) AFB_1_ standard with concentration of 100 ppb in liquid VY/2 medium. The peak at 8.27 min represents AFB_1_. (**B**) AFB_1_ treatment after 72 h incubation with culture supernatant. Peak at 3.17 min represents AFB_1_ biotransformation product.

**Figure 8:**
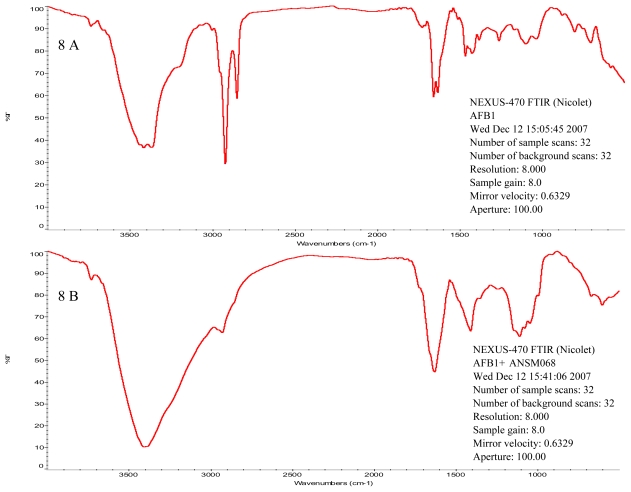
Infrared spectrometry spectrum of AFB_1_. (**A**) Spectrum of AFB_1_ standard. The initial AFB_1_ concentration was 100 ppb. (**B**) Spectrum of AFB_1_ product after 72 h incubation with culture supernatant. AFB_1_ biotransformation product was extracted by adding 2 mL chloroform to the incubation mixture and vortexing for 30 seconds.

Additionally, the HPLC chromatograph (Figure 7) confirmed that AFB_1_ (peak eluting at 8.27 min) was biotransformed to a new product eluting at 3.17 min. The IR spectrum showed that there were three major modifications in absorption peaks between the standard AFB_1_ and the product resulting from culture supernatant treatment (Figure 8). The absorption peak at 1728 cm^−1^ in AFB_1_ standard disappeared after the treatment, which indicates that the lactone attached to the benzene ring was modified. The two peaks in the area between 1658 and 1634 cm^−1^ in AFB_1_ standard changed into one peak after treatment. The change at 2930 cm^−1^ indicates modification of methyl group.

Alberts *et al*. [19] explored the AFB_1_ biotransformation product with *Rhodococcus erythropolis* extracellular fractions by electro spray mass spectrometry and liquid chromatography mass spectrometry analysis. By comparing the three distinct AFB_1_ peaks among treatments, AFB_1_ was still present but at a lower concentration. No breakdown products were revealed, which suggested that AFB_1_ was most likely metabolized to biotransformation products with chemical properties different from that of AFB_1._ Liu *et al*. [12] compared the infrared spectrum between AFB_1_ standard and fungal enzyme treated AFB_1_. Opening of the difuran ring was proposed since the group of five bands in the area between 1000 cm^−1^ and 1200 cm^−1^ did not appear in the spectrum of the enzyme treated AFB_1_. Marisa and Das [4] analyzed the biotransformed AFB_1_ structure by fluorescence spectra measurements and TLC. Treatment of AFB_1_ with purified enzyme resulted in a decrease in fluorescence intensity, suggesting the enzymatic cleavage of the lactone ring. The lactone ring was confirmed to be responsible for toxicity and mutagenicity in AFB_1_ [47,48]. In the current study, results from LCMS, HPLC and IR analysis confirmed AFB_1_ biotransformation by *M. fulvus* ANSM068 instead of cell binding. Modification of the lactone ring on the AFB_1_ molecule may result in detoxification although further investigation needs to be done. This result implies the potential applications of *M. fulvus* ANSM068 or its metabolites in detoxifying AFB_1_ in contaminated food or feed.

### 2.4. Characterization of Active Components from Culture Supernatant

**Figure 9:**
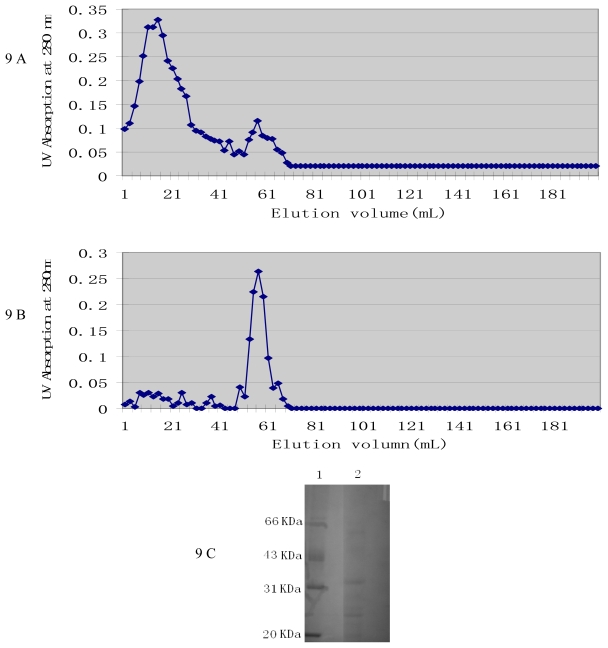
Determination of active components from culture supernatant by chromatography and SDS-PAGE. (**A**) Ion exchange chromatography on DEAE A-50 column. The column was pre-equilibrated with 50 mM sodium phosphate buffer pH 7.5. Elution was performed with the same buffer for the first 40 mL and then by a gradient of 0.5 M NaCl added to the buffer. Fractions of 2 mL were collected in tubes at a flow rate of 0.4 mL/min. (**B**) Molecular sieve chromatography on Sephadex G-100 column. The column was pre-equilibrated with 50 mM sodium phosphate buffer pH 7.5 containing 0.2 M NaCl. Proteins were eluted with the same buffer. Fractions of 2 mL were collected in tubes at a flow rate of 0.2 mL/min. (**C**) SDS-PAGE analysis of AFB_1_ biotransformation enzyme after purification by chromatography. Lane 1, Calibration marker proteins (Pharmacia LKB), 5 μL/lane; lane 2, peak fractions with AFB_1_ biotransformation activity from Sephadex G-100 chromatography, 20 μL/lane.

Since the culture supernatant of *M. fulvus* ANSM068 exhibited high AFB_1_ biotransformation activity, it was selected for active components extraction. Precipitates from 80% saturated ammonium sulfate showed higher AFB_1_ biotransformation activity, with 54.1% of AFB_1_ biotransformed. Chromatography was used to purify enzyme from the precipitates. Two peaks were obtained by ion exchange chromatography on DEAE A-50 column and UV detection at 280 nm (Figure 9A). The second peak, which showed AFB_1_ biotransformation activity (35.5%), was applied to molecular sieve chromatography on a Sephadex G-100 column (Figure 9B). SDS-PAGE showed that the active components consist of at least three proteins with a relatively small molecular weight between 20 and 66 kDa (Figure 9C).

To date, there are only a few reports on extraction or purification of mycotoxin biotransformation active components from microbial metabolites. Liu *et al*. [27] extracted and purified an intracellular enzyme from fungi *Armillariella tabescens* by ammonium sulfate precipitation, ion exchange chromatography and chromatofocusing chromatography. The AFB_1_ detoxification enzyme was then characterized to have a molecular mass of 51.8 kDa by SDS-PAGE and it exhibited a specific activity of 7.09 nmol min/mg at pH 6.0 and 28 °C. Motomura *et al*. [6] purified an aflatoxin biotransformation enzyme from the edible mushroom *Pleurotus ostreatus* by two chromatographies on DEAE-Sepharose and Phenyl-Sepharose. The apparent molecular mass of the purified enzyme was estimated to be 90 kDa by SDS-PAGE. This study has confirmed that the AFB_1_ biotransformation components exist in the culture supernatant and are very likely enzyme(s). Further purification and identification of the enzyme(s) are undergoing.

## 3. Experimental Section

### 3.1. Bacterium, Mycotoxin and Medium

*Myxococcus fulvus* ANSM068 was isolated and purified from deer feces collected from the Beijing Zoo in July, 2006. It was identified using physiological and biochemical tests and 16S rRNA gene sequence analysis by the China General Microbiological Culture Collection Center (CGMCC) and has been deposited there as CGMCC #3194. It was preserved at −80 °C before use (China Agriculture University, Beijing, China).

AFB_1_ was obtained from Sigma Chemical Co. (Bellefonte, USA). The medium used for liquid culture, VY/2 medium, contains 5 g yeast extract, 1 g CaCl_2_, 0.5 g MgSO_4_, 0.5 mg VB_12_ in 1 L distilled water at room temperature.

### 3.2. AFB_1_ Quantification

For AFB_1_ analysis, the HPLC procedure by AOAC [49] was used with slight modifications. The reaction mixtures were extracted three times with chloroform. The chloroform extracts were evaporated under nitrogen at room temperature, the residue was dissolved in 50% methanol in water (1:1, v/v) and analyzed by HPLC. HPLC analysis was performed using a LiChroCART RP-C18 (250-4 Hypersil ODS (5 μm), Merck) column with a guard column (LiChroCART 4-4 RP-C18 (5 μm), Merck). The mobile phase was methanol: water (1:1, v/v) isocratic at a flow rate of 1 mL/min. AFB_1_ was derivatized by a photochemical reactor (AURA, USA) and measured by a fluorescence detector. The excitation and detection wavelengths were set at 360 and 440 nm, respectively. The percentage of AFB_1_ biotransformation was calculated using the following formula:

$$(1-{\text{AFB}}_{1}\;\text{peak area in treatment}/{\text{AFB}}_{1}\;\text{peak area in control})\times 100\%$$

### 3.3. Efficacy of AFB_1_ Biotransformation by *M. fulvus* ANSM068

Biotransformation of AFB_1_ by *M. fulvus* ANSM068 was carried out in liquid culture. The bacterial isolate was cultured in VY/2 medium for 24 h and used to inoculate VY/2 (50 mL) in a 300 mL flask, which was incubated at 30 °C with agitation at 160 rpm for 50 h in a Gyrotary shaker incubator without AFB_1_ (Haerbin Donglian Electronic Equipment Inc., China). AFB_1_ standard solution (Sigma Chemical Co., Bellefonte, USA) was diluted with methanol (Beijing Chemical Inc., Beijing, China) to a stock solution of 500 ppb, of which 0.2 mL was added to bacterial cultures of 0.8 mL for a final concentration of 100 ppb. The biotransformation tests were conducted in the dark at 30 °C without shaking for 72 h. After incubation, bacterial cells were removed by centrifugation at 9,300 g for 10 min (TGL-16C centrifuge, Beijing Medical Centrifuge Inc., China). Sterile VY/2 medium was used to substitute the bacterial culture in the control.

Biotransformation of AFB_1_ by cells, culture supernatant and intracellular cell extracts was conducted according to the procedure of Shu *et al*. [22]. Cells were pelleted using a centrifuge at 9,300 g, 4 °C for 10 min. The pellets were washed twice with a phosphate buffer (50 mM; pH 7.5) before being resuspended in the phosphate buffer (5 mL). Culture supernatant was obtained by centrifuging 50 h liquid culture at 9,300 g, 4 °C for 10 min. Intracellular cell extracts were harvested by the following procedures. The cell suspension was disintegrated twice (work every other 5 s for 33 min) by using ultrasonic cell disintegrator on ice. The disintegrated cell suspension was centrifuged at 9,300 g for 20 min at 4 °C. The cell extracts were collected by filtering the supernatant aseptically using 0.2 μm pore size sterile cellulose pyrogen free filters. The phosphate buffer solution (50 mM; pH 7.5) was used to substitute intracellular cell extracts and cells in the controls. The effect of protease treatment was determined by exposing the culture supernatant to 1 mg/mL proteinase K (Roche Diagnostics, Basel, Switzerland; specific activity ≥ 30 U/mg) for 1 h at 37 °C; 1 mg/mL proteinase K plus 1% SDS for 6 h at 37 °C. The effect of heat treatment was determined by dipping the culture supernatant into a boiling water bath for 10 min. 0.2 mL of AFB_1_ stock solution was added to 0.8 mL of each reaction liquid for a final concentration of 100 ppb. The biotransformation tests were conducted in the dark at 30 °C without shaking for 72 h. After incubation, bacterial cells were removed by centrifugation at 9,300 g for 10 min (TGL-16C centrifuge, Beijing Medical Centrifuge Inc., China). The untreated culture supernatant was used as control.

### 3.4. Culture Conditions Affecting AFB_1_ Biotransformation

Unless specifically indicated, all biotransformation experiments were conducted by using culture supernatant at 30 °C for 72 h with aeration. Culture supernatant was obtained by centrifuging 50 h culture with 9,300 g at 4 °C for 20 min.

To detect the appropriate nitrogen source for the bacterial growth with optimum AFB_1_ biotransformation, 0.5% of tryptone, peptone, beef extract, yeast extract, fish peptone and ammonium nitrate (NH_4_NO_3_) were chosen as the nitrogen source in the culture medium.

Initial pH value in VY/2 medium was adjusted to 5.0, 5.5, 6.0 by using citrate acid buffer, and to 6.5, 7.0, 7.5, 8.0, 9.0 by sodium phosphate buffer.

Cultivation was performed at 15, 20, 25, 30, 35 and 40 °C respectively for 50 h before the culture supernatants were harvested.

### 3.5. AFB_1_ Biotransformation Product Analysis

The biotransformation products of AFB_1_ after 72 h incubation with culture supernatants were determined by liquid chromatography mass spectrometry (LCMS) and infrared analysis (IR). For LCMS, the following samples were analyzed: (a) AFB_1_ standard, (b) VY/2 medium supplemented with AFB_1_, (c) AFB_1_ treated with culture supernatants for 72 h. The samples were extracted with chloroform, dried under nitrogen, suspended in methanol: water (1:1, v/v) and analyzed by LCMS using a Phenomenex 2.0 × 150 mm C_18_ column and methanol: acetonitrile: water (1:1:2, v/v/v) as solvent, at a flow rate of 100 μL/min. The HPLC system was equipped with a Finnigan Spectra System UV6000LP ultraviolet (UV) detector. Atmospheric pressure chemical ionization (APCI) positive ion mode was used for MS detection (ThermoFinnigan, San Jose, CA, USA).

For IR analysis, 2 mL chloroform was added to the incubation mixture and vortexed for 30 seconds. The phases were separated by low-speed centrifugation and the organic phase was evaporated to dryness at 20 °C under N_2_. The samples were purified by HPLC before IR analysis. IR analysis was carried out with a KBr pellet using an IR spectrophotometer (NEXUS-470F TIR, Nicolet, Japan). Sterile VY/2 medium was used to substitute culture supernatant in the control.

### 3.6. Determination of Active Components from Culture Supernatant

#### 3.6.1. Precipitation of Active Components

Methanol, acetone, ethanol and solid ammonium sulphate were used to precipitate the active components. For the organic solvents, 500 mL of each of the chilled solvent was added to 150 mL of the culture supernatant. For ammonium sulphate, 150 mL of the culture supernatant was supplemented to 20, 40, 60 and 80% saturation respectively under constant stirring. The above solutions were stored at 4 °C for 4 h followed by centrifugation at 9,300 g for 20 min. The precipitates were dissolved in 2 mL of 50 mM sodium phosphate buffer (pH 7.5). The ammonium sulphate treatments were dialysed over night against distilled water before being applied to chromatography.

#### 3.6.2. Purification of the Active Components by Chromatography

The dialyzed samples were applied to a DEAE A-50 Ion exchange column (Pharmacia Biotech, Sweden) pre-equilibrated with 50 mM sodium phosphate buffer pH 7.5. Elution was performed with the same buffer and by a gradient of 0.5 M NaCl in the buffer. Fractions of 2 mL were collected in separate tubes at a flow rate of 0.4 mL/min. The protein concentration in each eluted fraction was detected at 280 nm using a UV-visible spectrophotometer. The fractions with AFB_1_ biotransformation activity were combined and applied to a Sephadex G-100 Molecular sieve column (Pharmacia) pre-equilibrated with 50 mM sodium phosphate buffer containing 0.2 M NaCl. Proteins were eluted with the same buffer and fractions of 2 mL were collected in separate tubes at a flow rate of 0.2 mL/min. After dialysis against buffer, the AFB_1_ biotransformation activity of each fraction was tested.

#### 3.6.3. Sodium Dodecyl Sulphate-Polyacrylamide Gel Electrophoresis (SDS-PAGE)

SDS-PAGE was performed in 12.5% polyacrylamide gels according to the method of Motomura *et al*. [6]. The separated proteins were stained with Coomassie Brilliant Blue R-250 (Fluka, Switzerland) and their molecular weights were determined by comparison with low range molecular weight markers (Pharmacia Biotech, Uppsala, Sweden).

### 3.7. Statistical Analyses

The results were average of triplicates and were expressed as mean ± standard error. Data were analyzed as a completely randomized single factor design by ANOVA using the general linear models procedure in SAS. Significant F tests at the 0.05 levels of probability are reported. When a significant F-value was detected, Duncan’s Multiple Range Test was used to determine significant differences among means.

## 4. Conclusions

The present study demonstrated the ability of *M. fulvus* ANSM068 to effectively biotransform AFB_1_. Furthermore, optimal conditions for biotransformation were determined as follows: 0.5% of yeast extract as nitrogen source; initial pH value at 7.5; temperature at 30 °C. The results indicated that enzyme(s) in the culture supernatant was responsible for the biotransformation although further confirmation is needed. Research is underway to purify the effective enzyme(s) and to identify metabolites produced during the biotransformation processes. The AFB_1_ biotransformation enzymes, once identified, may be mass-produced by the bacterial isolate and be used to treat materials contaminated with AFB_1_.
